# Long-term dynamics of urban thermal comfort in China’s four major capital cities across different climate zones

**DOI:** 10.7717/peerj.8026

**Published:** 2019-11-11

**Authors:** Yao Fu, Zhibin Ren, Qiuyan Yu, Xingyuan He, Lu Xiao, Qiong Wang, Chang Liu

**Affiliations:** 1 Key Laboratory of Wetland Ecology and Environment, Northeast Institute of Geography and Agroecology, Chinese Academy of Sciences, Changchun, Jilin, China; 2 University of Chinese Academy of Sciences, Beijing, China; 3 Plant and Environment Sciences, New Mexico State University, Las Cruces, NM, United States of America; 4 College of Forestry, Southwest Forest University, Kunming, Yunnan, China

**Keywords:** Urban thermal comfort, Urbanization, Urban planning

## Abstract

China has experienced intensive urbanization over the past decades. However, it is still unclear about the influence of urbanization on urban thermal comfort and how the effect varies with climate condition. Based on long-term daily meteorological data from 1990 to 2015 in four Chinese cities undergoing rapid urbanization, our study tried to detect the long-term dynamics of summer urban thermal comfort across different climate zones and analyze their relationships with urbanization. Our results showed that urbanization can increase urban temperature and decrease relative humidity and wind velocity. Urban thermal comfort and discomfort days also changed greatly, especially in Harbin, Northeast China from 1990 to 2015. However, such changes for different cities across different climate zones are inconsistent. Results also showed that urbanization especially for social economic activities can have a significant influence on the physiological Equivalent Temperature (PET). Compared with southern cities, the PET in northern cities such as Harbin and Changchun in this study is more sensitive to urbanization. These results reveal that the changing patterns of urban thermal comfort in Chinese cities under rapid urbanization, and help government take some effective measures to improve urban thermal environment.

## Introduction

With the intensive development of urbanization, urban thermal environment is becoming vital to human population as it affects the general well-being. It affects the performance and operations of the inhabitants and to large extent as well bring about health issues especially for elder individuals. Understanding the thermal environmental condition is important as it helps in promoting awareness and the development of efficient management strategies (Feyisa, Dons & Meilby, 2014; Hajat, Kovats & Lachowycz, 2007; Kuang, 2019; Li et al., 2018; Tan et al., 2010). Urban thermal environment is a particular concern on hot summer days when intensive thermal heat increases the need for air conditioning in buildings, and causes discomfort to people, both indoors and outdoors. It is likely to get worse with climate change, as mean temperatures are predicted to rise, as are intensity of extreme weather such as heat waves (Armson, Stringer & Ennos, 2012; Cao et al., 2018; Mladenovic et al., 2016).

Evaluation of urban thermal environment has been mainly conducted with two types of temperature data (Eludoyin et al., 2014; Fu & Weng, 2016; Mushore et al., 2017; Qiao, Tian & Xiao, 2013), (1) land surface temperature (LST) derived from remotely sensed thermal infrared (TIR) imagery; and (2) *in-situ* air temperature collected from meteorological stations. Unfortunately, temperature measurements barely reflects human perception of thermal comfort (Fanger, 1972; Ketterer & Matzarakis, 2014). Urban thermal comfort is a state of mind that articulates satisfaction with the urban thermal environment depending on the combined effect of the physical and climatic parameters (Aljawabra & Nikolopoulou, 2018; Galagoda et al., 2018). In such sense, human thermal comfort can be measured as a synthesis of meteorological parameters such as air temperature, relative humidity, and wind velocity. It describes the urban thermal environment in a thermophysiologically weighted way (Höppe, 1999). Evaluating the long-term dynamics of urban thermal comfort is essential for appropriate urban planning as the achievement of urban planning design lies on the thermal comfort perception by urban residents (Ndetto & Matzarakis, 2013).

China has experienced rapid urbanization in recent decades, and about 60% of its total population would live in urban areas by 2020, rapid urbanization in China has accompanied a surge in population, expansion of urban footprints, and economic development (Mao et al., 2018; Zhou & Chen, 2018). Ongoing rapid urbanization has the potential to improve the well-being of Chinese societies. Yet, the accelerated urbanization also presents challenges to maintain thermal comfort increasing (Mladenovic et al., 2016; Morris et al., 2017). Improving urban thermal environment is vital in China, as major cities in China are faced with the challenge of unplanned urban development (He et al., 2017; Yao et al., 2017). Many studies suggest that surface modification factors, such as the expansion of built-up areas or the decline in vegetation coverage, affect urban climate (Cao et al., 2018; Mahmoud & Gan, 2018; Xiong et al., 2012). Urbanization processes accompany with the increases of artificial surface and anthropogenic heat release, which induces the modification of surface energy partitioning and urban energy balance, this changes the thermal characteristics of the underlying surface. Studies have shown significant relationships between urbanization and urban temperature, Yao et al. (2017) analyzed trends of surface urban heat islands and associated determinants in major Chinese cities, and found that reduction in vegetation and increasing anthropogenic heat release contributed to the increasing surface urban heat islands intensity in urbanized areas. Fu & Weng (2016) made a time series analysis of urbanization impact on LST, and found that the conversion of evergreen forest to medium-intensity urban land generated the largest difference in annual LST variation and the largest trend difference. Lin et al. (2018) showed spatiotemporal thermal patterns in Hangzhou that heat energy spill from the urban land to the natural land during urbanization processes. Wang et al. (2016) found that urban expansion would lead to an increase of 5 K for the minimum temperature in the newly developed areas. However, how urbanization affects urban thermal comfort is still not well-understood. To accommodate the rapid expansion in urban dwelling, we need to understand the impacts of urbanization on urban thermal comfort. Mahmoud & Gan (2018) analyzed trend of three thermal comfort indexes in the Cairo governorate of Egypt, and the research of Wu, Yang & Shen (2019) investigated regional variations of outdoor thermal comfort in China from 1966 to 2016. But further research of the relationship between urban thermal comfort dynamic and urbanization factors is needed. It is well known that each city is embedded in a specific regional atmospheric situation, which is determined by its respective climate zone and topographic conditions (Holst & Mayer, 2011; Lee, Mayer & Chen, 2016). There is, therefore, no one-size-fit-all urban strategy to enhance thermal comfort. Understanding the spatiotemporal dynamics of urban thermal environments and comfort of varying climatic condition under China’s rapid urbanization is essential as such knowledge can be applied by urban designers and planners for different cities, in the constitution of suitable and sustainable designs.

This study, therefore, proceeds to assess the thermal comfort condition of four Chinese cities with varying climatic environment. Specifically, the study aims to: (1) detect the spatiotemporal changes of urban climate in summer under rapid urbanization for a long-term period of 26 years in different climate zones; and (2) explore the spatiotemporal changes of urban thermal comfort in summer for these cities and analyze the relationship with urbanization factors.

## Materials & Methods

### Study areas

**Figure 1:**
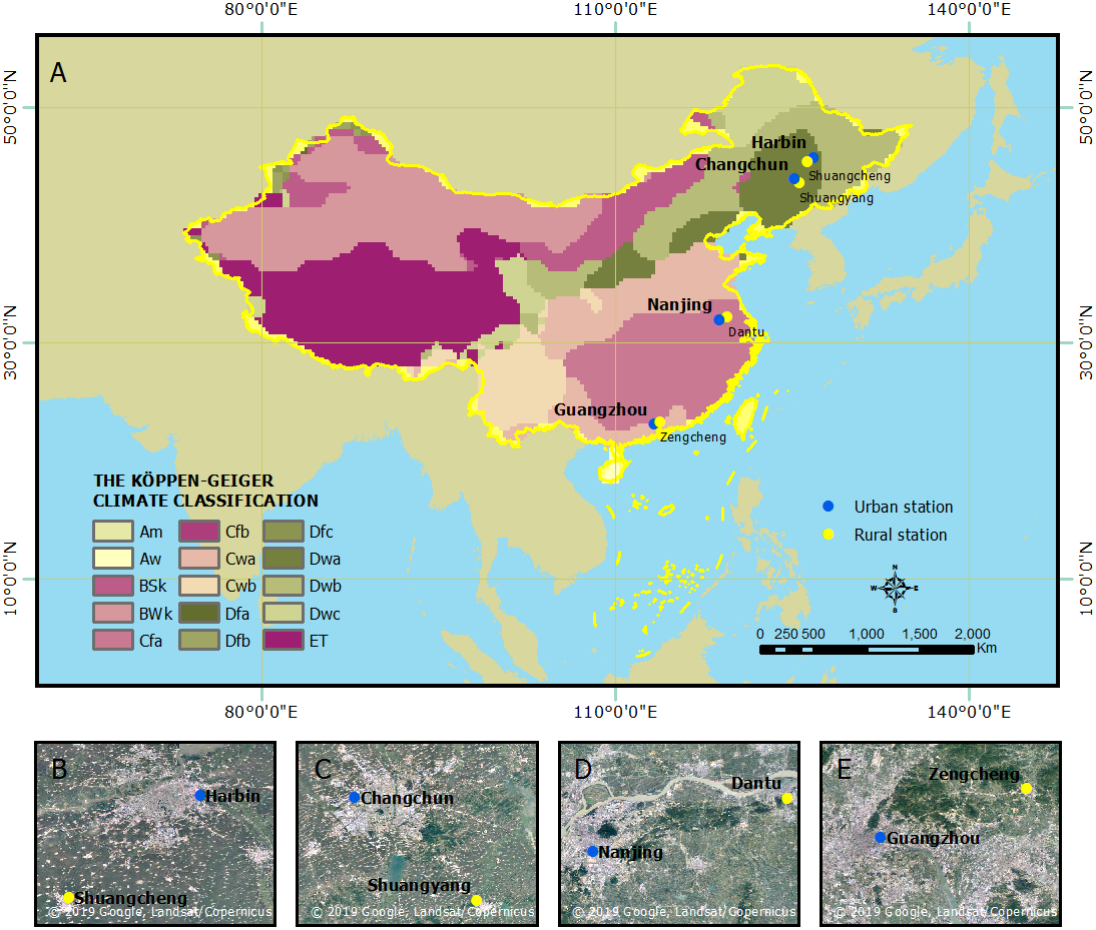
Spatial distribution of cities in this study. (A) Location of all the cities; (B) Harbin; (C) Changchun; (D) Nanjing; (E) Guangzhou. Map data © 2019 Google, Landsat/Copernicus.

The study was carried out in four cities: Harbin, Changchun, Nanjing, and Guangzhou in two different climate zone (Fig. 1). Harbin and Changchun have a humid continental climate (Dwa) with a mean annual temperature of 5.8 °C and annual precipitation of 546.2 mm, the mean annual relative humidity is 62.8%. Nanjing and Guangzhou experience humid subtropical climate (Cfa) with a mean temperature of 19.4 °C and annual precipitation of 1488.7 mm, the mean annual relative humidity is 74.2%. The climatology attributes of these cities are based on Köppen-Geiger (Kottek et al., 2006).

### Data collection

#### Urban thermal comfort

The assessment of thermal comfort was done by computing the Physiological Equivalent Temperature (PET) for each selected city. PET is a widely used thermal index derived from human energy balance. It is well suited to the evaluation of thermal condition of different climates (Potchter et al., 2018). PET presents the thermal condition of the human body in a physiologically relevant way with air temperature, relative humidity, wind velocity, and solar radiation is preferable to other thermal indices because of its unit (°C), which make results more comprehensive to people and urban planners (Matzarakis, Mayer & Iziomon, 1999).

The meteorological variables (temperature, relative humidity, wind velocity and radiation) were obtained from Dataset of daily climate data from Chinese surface stations (V3.0) and Dataset of daily radiation data, China Meteorological Data Service Center (http://data.cma.cn), covering 26 years of daily measurements over summer (June, July, and August) from 1990 to 2015.

PET index of urban stations was calculated by RayMan model, which is a micro-scale model, it requires only basic meteorological data for the calculation of radiation fluxes and common thermal indices for the thermal human-bioclimate (Matzarakis, Rutz & Mayer, 2007; Matzarakis, Rutz & Mayer, 2010). We also used the length of summer discomfort period regarding to the days with PET above 30 °C, because people would suffer from moderate, strong, or extreme heat stress in these days according to Table 1 (Matzarakis, Mayer & Iziomon, 1999). The thermophysiological parameters of the people in the calculation of PET were those of a typical male (35 years old, 1.75 m height, 75 kg weight) with an internal heat production of 80 W and a heat transfer resistance of the clothing of 0.9 clo.

**Table 1:** Thermal sensation classes for human beings.

**PET (°C)**	**Thermal perception**	**Grade of physical stress**
>42	Very hot	Extreme heat stress
36–42	Hot	Strong heat stress
30–36	Warm	Moderate heat stress
24–30	Slightly warm	Slight heat stress
18–24	Comfortable	No thermal stress
13–18	Slightly cool	Slight cold stress

To explore the relationship between urban thermal comfort and urbanization, the effect of the background climate had to be separated. We used only two meteorological stations for each city, an urban station and a rural station (Table 2), because observations of meteorological data are typically only available from a limited number of weather stations. The thermal environment is calculated using the data collected by the urban stations, while data from rural stations are used to represent background climate conditions. The urban stations are located inside the urban areas, and the distance between the stations to the urban center is less than 10 kilometers, surrounding areas of urban stations are dominated by mid-rise and high-rise buildings. The distance from rural station to urban station of each city was more than 50 kilometers, surrounding areas of rural stations are dominated by natural land cover such as farmland. Rural stations have no radiation record which is essential to calculate PET. Since air temperature and PET are highly correlated, therefore, the temperature of rural stations was used as a proxy of PET of background.

**Table 2:** Geographic and climatic characteristics of the synoptic stations used in the study.

**Station**	**Longitude**	**Latitude**	**Altitude (m)**	**Distance (km)**	**Mean annual temperature (1990∼2015) (°C)**	**Mean annual precipitation (1990∼2015) (mm)**	**Mean annual relative humidity (1990∼2015) (%)**	**Climate zone**
**Harbin**	126°34′E	45°56′N	118	77	5.2	522.8	64.1	Humid-continental zone (Dwa)
Shuangcheng	126°19′E	45°24′N	166		5.1	496.2	64.9	
**Changchun**	125°13′E	43°54′N	236	78	6.4	569.6	61.5	
Shuangyang	125°38′E	43°33′N	219		6.0	622.6	65.3	
**Nanjing**	118°54′E	31°56′N	35	78	16.3	1112.4	73.2	Humid-subtropical zone (Cfa)
Dantu	119°28′E	32°11′N	28		16.1	1114.7	72.5	
**Guangzhou**	113°29′E	23°13′N	70	59	22.5	1864.9	75.1	
Zengcheng	113°50′E	23°20′N	31		22.0	1996.1	78.4	

**Notes.**

Stations in bold are urban stations; the other stations are rural stations. Distance: distance between urban station to rural station.

#### Urbanization indices

Rapid urbanization in China has accompanied by a surge in population, expansion of urban footprints, and economic development. Urbanization indices characterizing population growth and economic development were employed in our study: population, GDP (108 yuan), primary industry (108 yuan), secondary industry (108 yuan), and tertiary industry (108 yuan) (Urban statistical yearbook of China, 1990–2015).

### Data analysis

Daily temperature(Ta), relative humidity(RH), wind velocity(Wind), global radiation(Ra), and the PET in each city were averaged through summer. The time series of discomfort days (D days) were computed as the length of discomfort period of each summer.

#### Temporal trend analysis of urban thermal environment and comfort

We conducted the Mann-Kendall trend test and Sen’s slope estimator nonparametric test for air temperature, relative humidity, wind velocity, radiation, thermal comfort index(PET), and discomfort days in summer to detect the temporal trends of meteorological and thermal comfort series through 1990 to 2015. These methods have been widely used in the trend analysis of hydrological and climatologic variables (Bari et al., 2016; Gocic & Trajkovic, 2013). These two tests are preferred than parametric tests due to their flexibility of using hypotheses for variables (Bari et al., 2016; Partal & Kahya, 2006; Shifteh Some’e, Ezani & Tabari, 2012; Yue, Pilon & Cavadias, 2002). The Mann-Kendall’s trend test was used to capture trends of urban climate and thermal comfort. A positive value of Mann-Kendall estimator Zs indicates an upward trend, while negative Zs implies downward trend. For cities with significant trend of urban climate and thermal comfort, the change rate was obtained by the Sen’s slope.

A modified pre-whitening method Trend Free Pre-Whitening (TFPW) was used to remove serial correlation from the series when there was a significant serial correlation (Shifteh Some’e, Ezani & Tabari, 2012; Yue & Wang, 2002). The lag-1 serial correlation coefficient (r1) of the series was calculated using (Gocic & Trajkovic, 2013). If r1 is not significant, the sample data are considered serially independent; then the Mann-Kendall test is applied to the original values of the time series. Otherwise, it is serially correlated, the TFPW method is used before the application of the Mann-Kendall test.

#### The change point of urban thermal comfort series

We also detected the change point of urban thermal comfort from 1990 to 2015 using the Mann-Kendall rank statistic test with the series of PET and discomfort days in summer. This test sets up two series, *u*(*t*) and *u*′(*t*); a progressive one and a backward one. When the value of *u*(*t*) is significant at a desired level of significance, one can decide whether it is an increasing or decreasing trend depending on whether *u*(*t*) > 0 or *u*(*t*) < 0 (Türkeş, 1996). If they cross each other and diverge beyond the specific threshold value (95% confidence limit in the present analysis), then there is a statistically significant change point (Bari et al., 2016). The point where they cross each other indicates the approximate year at which the trend begins (Mosmann et al., 2004).

#### Urbanization factors associated with temporal variations of the PET

To explore the effects of urbanization on temporal variations of the PET, we conducted stepwise regression analysis between the PET and urbanization variables (urban population, GDP, primary industry, secondary industry, and tertiary industry) over the studies years for each city. The temperature of rural stations (T-rural) was used as a reference to present background climate conditions, assuming that there is no urbanization effect on rural climate. The PET was used as a dependent variable, and the urbanization variables and T-rural were used as independent variables. All statistical analyses were performed in SPSS Statistics 21.0 (IBM, Armonk, NY, USA).

## Results

### Long-term dynamics of meteorological parameters in different cities

The time series analysis of meteorological parameters suggests an overall warming trend for most cities except for Guangzhou. The Zs are 2.265, 1.565, 1.425, and −0.304 for Harbin, Changchun, Nanjing, and Guangzhou, respectively (Table 3). However, only Harbin has a statistically significant upward trend at the 5% significance level. Based on the Sen’s slopes estimator, the significant increasing rates of Ta was 0.052 °C per year at Harbin. Urban relative humidity and wind velocity have also changed dramatically during the past 26 years. According to the Mann-Kendall test, all cities exhibited significant downward trend in relative humidity except for Harbin city. The magnitudes of the significant downward trends in relative humidity ranged between 0.147% per year at Guangzhou and 0.274% per year in Changchun. Wind velocity appeared to increase in Nanjing and Guangzhou station, and Nanjing has a statistically significant upward trend. The remaining cities have significant negative trends in wind velocity. The magnitudes of the significant downward trends in wind velocity ranged between 0.029 m/s per year at Changchun and 0.044 m/s per year at Harbin. Guangzhou presents significant increasing trends in the global radiation series.

**Table 3:** The statistical tests for summer meteorological parameters from 1990 to 2015.

**Variable**	**Test**	**Harbin**	**Changchun**	**Nanjing**	**Guangzhou**
Ta	Zs	2.265^*^	1.565	1.425	−0.304
	Qmed	0.052	0.039	0.022	−0.008
	r1	0.208	0.008	−0.029	**0.404**
RH	Zs	−1.215	−2.873^**^	−2.056^*^	−2.592^**^
	Qmed	−0.169	−0.274	−0.217	−0.147
	r1	0.297	0.033	0.251	**0.633**
Wind	Zs	−4.414^**^	−3.480^**^	3.246^**^	0.864
	Qmed	−0.044	−0.029	0.020	0.006
	r1	**0.553**	**0.514**	**0.575**	**0.495**
Ra	Zs	−0.257	−0.958	−0.771	3.807^**^
	Qmed	0.125	−0.810	−0.500	3.052
	r1	−0.001	−0.172	**−0.391**	0.317

**Notes.**

Zs Mann-Kendall estimator Qmed Sens slope estimator r1 Lag-1 serial correlation coefficient (serial correlation are presented in bold character) Ta temperature RH relative humidity Wind wind velocity Ra global radiation

* Statistically significant trends at the 5% significance level.

** Statistically significant trends at the 1% significance level.

### Long-term dynamics of PET and discomfort days in different climate zones

**Figure 2:**
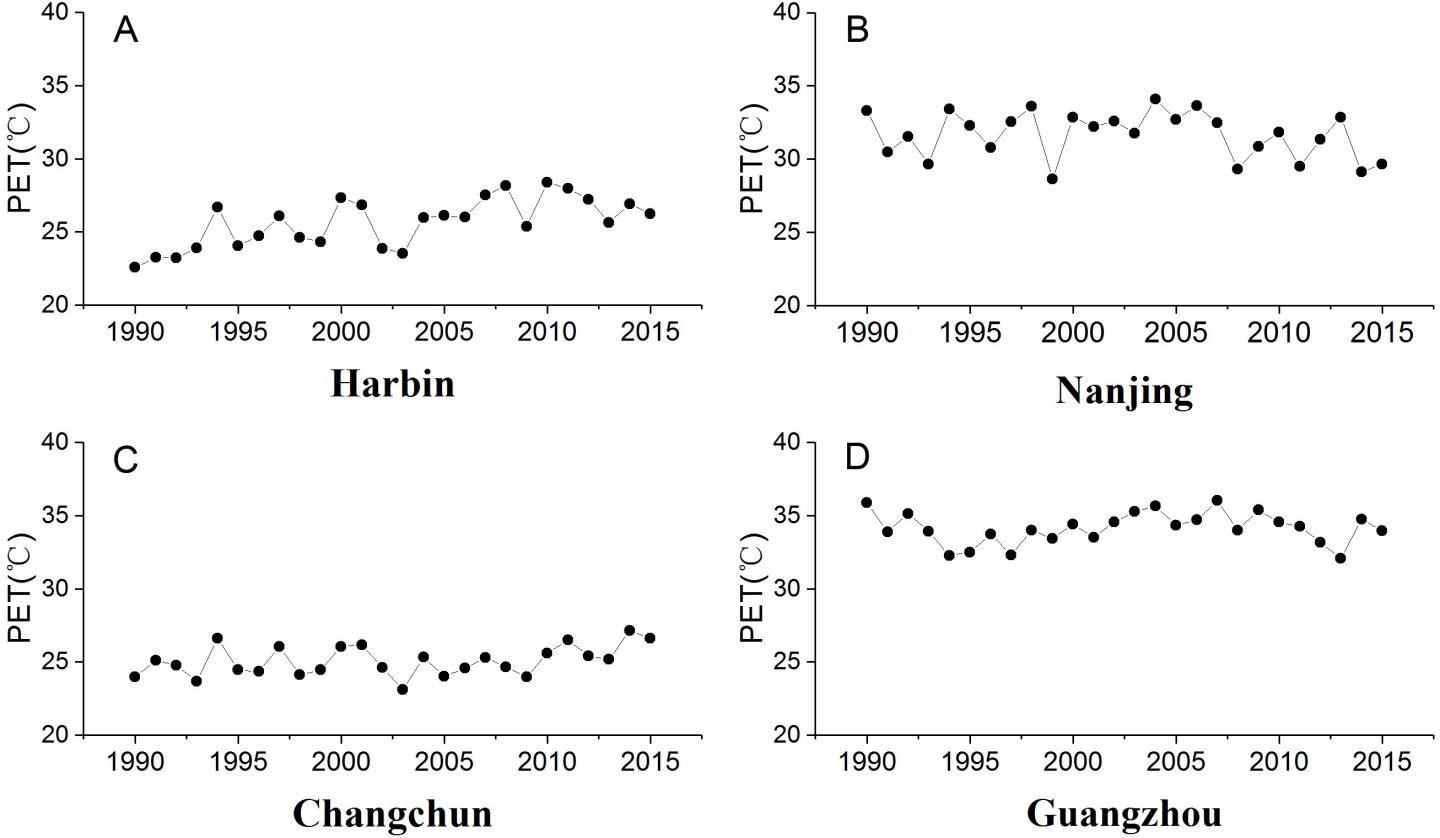
Trends of summer PET (°C) of urban stations in different climate zones. (A) Harbin, (B) Nanjing, (C) Changchun, (D) Guangzhou.

**Figure 3:**
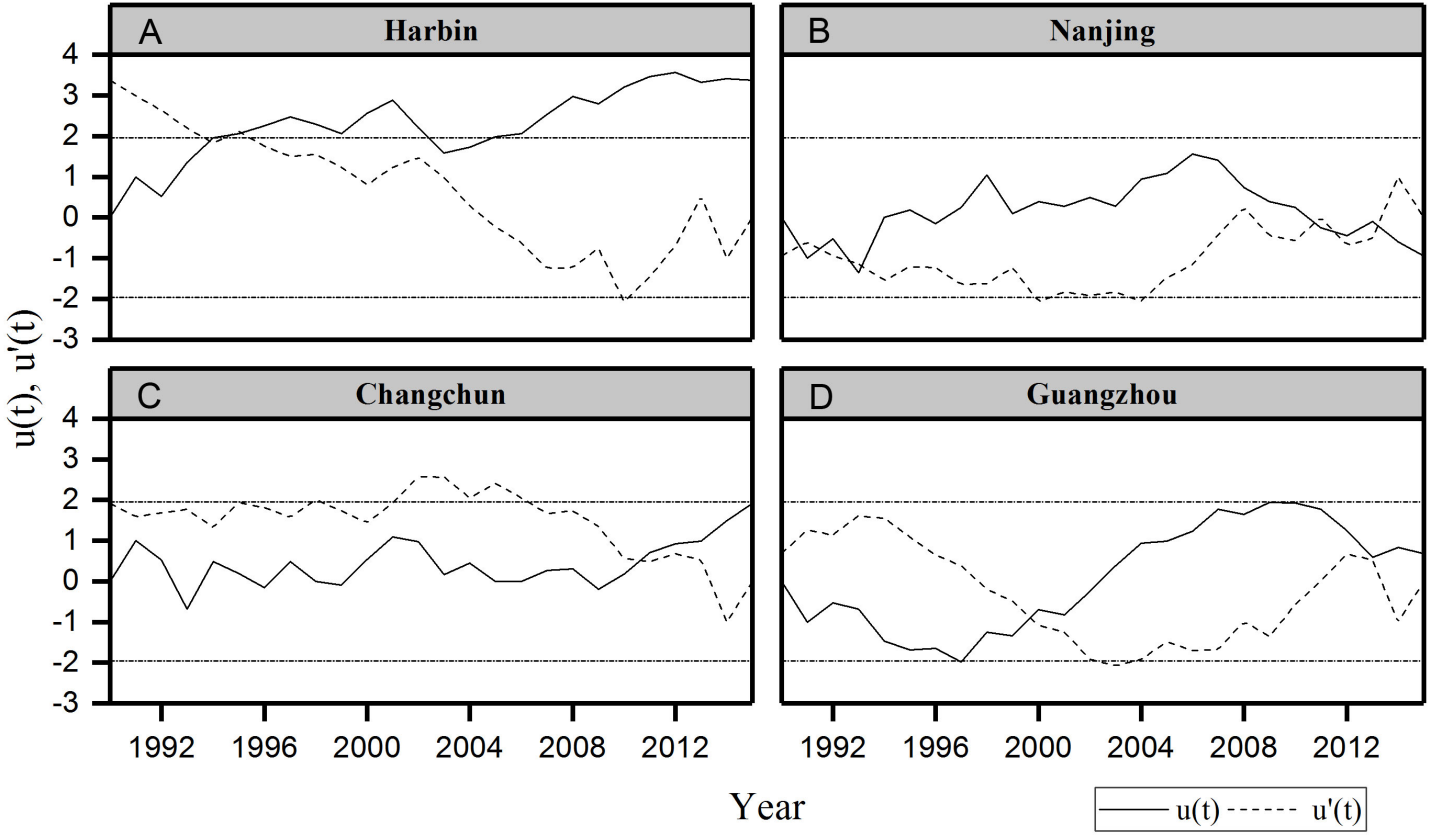
Sequential Mann–Kendall test rank statistics for summer PET. (A) Harbin, (B) Nanjing, (C) Changchun, (D) Guangzhou. Dotted horizontal straight lines indicate the lower limit and upper limit of 95% confidence intervals.

Summer urban thermal comfort varied significantly from 1990 to 2015 (Fig. 2). The summer PET in Harbin and Changchun are generally less than 30 °C during these years. The corresponding thermal perception is slightly warm or comfortable, which equates to slight heat stress or no thermal stress (Table 1). The PET in summer at Nanjing and Guangzhou are mostly between 30∼36 °C, and the corresponding thermal perception is warm with moderate thermal stress for the corresponding grade of physical stress (Table 1). Urban thermal comfort also changed greatly during the last 26 years in the four cities. Our results showed an increasing trend of summer PET for all the cities except Nanjing (Table 4). However, only the increasing trend of PET for Harbin in Northeast China was statistically significant. Besides, Harbin city also had the highest PET increasing rate, about 0.158 °C per year. In addition, a significant change point was identified for Harbin in 1994 (Fig. 3). The *u*(*t*) and *u*′(*t*) statistics of the sequential Mann-Kendall rank statistic test experienced non-significant change point for the other cities. Although it is not significant, an increasing trend was observed in Changchun and Guangzhou after 2010 and 2000 respectively. Moreover, a slightly decrease was observed at Nanjing after 2011.

**Table 4:** The statistical tests for PET and discomfort days over 1990–2015.

**Variable**	**Test**	**Harbin**	**Changchun**	**Nanjing**	**Guangzhou**
PET	Zs	4.040^**^	1.565	−0.584	1.238
	Qmed	0.158	0.050	−0.049	0.016
	r1	**0.447**	0.064	−0.057	0.271
D days	Zs	3.620^**^	1.150	−1.078	1.883
	Qmed	0.769	0.200	−0.429	0.231
	r1	**0.385**	0.109	−0.039	0.106

**Notes.**

Zs Mann-Kendall estimator Qmed Sens slope estimator r1 Lag-1 serial correlation coefficient (serial correlation are presented in bold character) D days discomfort days in summer

* Statistically significant trends at the 5% significance level.

** Statistically significant trends at the 1% significance level.

**Figure 4:**
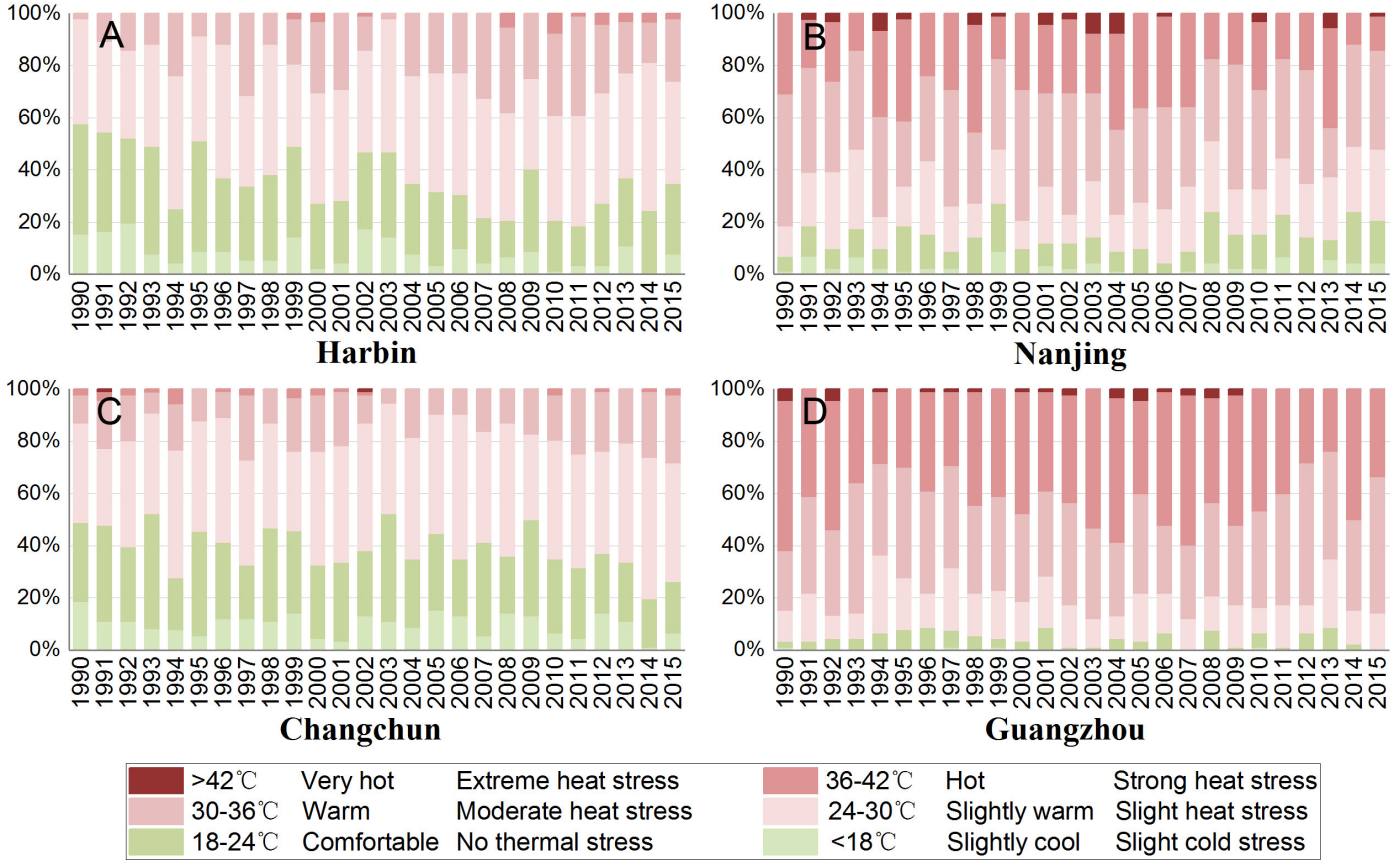
Percent stacked column of summer thermal comfort grades from 1990 to 2015. (A) Harbin, (B) Nanjing, (C) Changchun, (D) Guangzhou.

**Figure 5:**
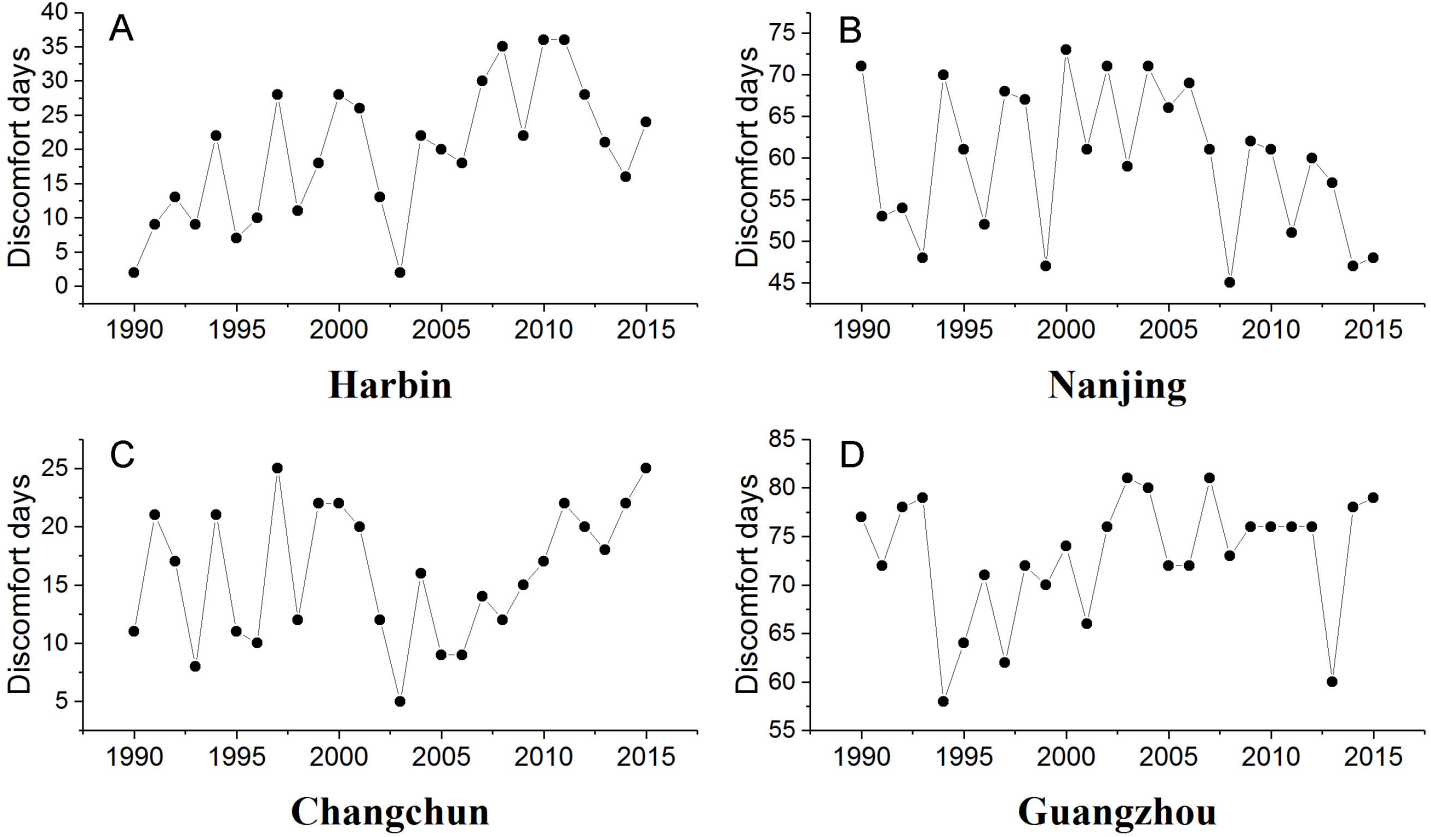
Trends of discomfort days of urban stations in different climate zones. (A) Harbin, (B) Nanjing (C) Changchun, (D) Guangzhou.

**Figure 6:**
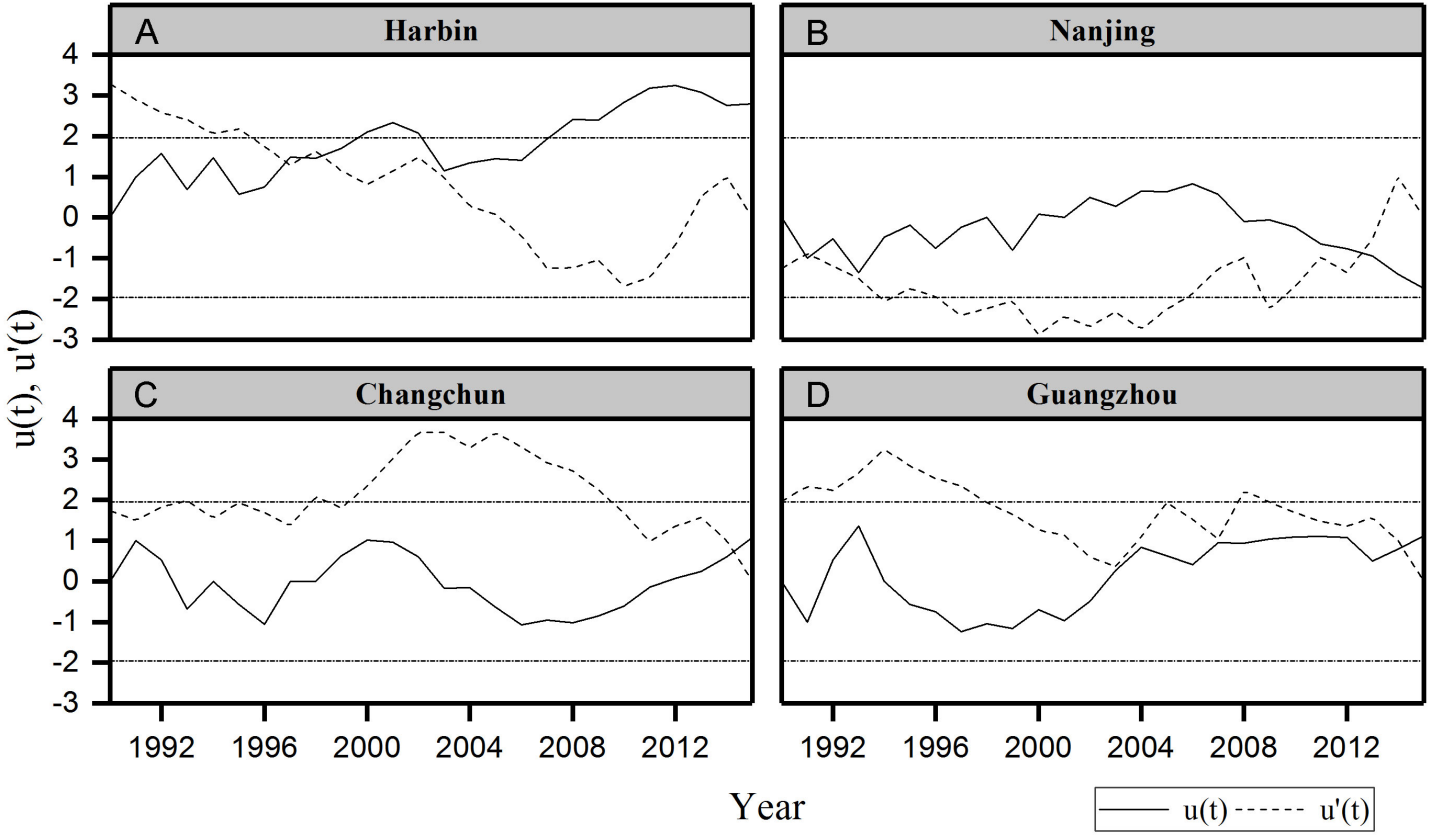
Sequential Mann–Kendall test rank statistics for discomfort days in summer. (A) Harbin, (B) Nanjing (C) Changchun, (D) Guangzhou. Dotted horizontal straight lines indicate the lower limit and upper limit of 95% confidence intervals.

Figure 4 demonstrates the frequency distribution of PET in summer for different cities. The results showed that most of the summer days in Harbin and Changchun are comfortable or slightly warm. Harbin and Changchun had more comfortable summers with longer comfortable period in summer than cities in subtropical regions. The PET of most days in summer is 30∼36 °C in Nanjing. The corresponding thermal perception is warm and the grade of the physical stress is moderate heat stress. Guangzhou is the hottest among these cities. Most summer days of Guangzhou are hot or warm, suggesting that people suffer from strong heat stress or moderate heat stress during the summer period, which makes people feel extremely uncomfortable. In addition, we also found that urban discomfort days increased during the last 26 years. Our results showed that there are upward trends of urban discomfort days for all cities except for Nanjing (Fig. 5). The discomfort days of Harbin have extended about 0.769 day per year (Table 4). For Harbin, a long-term increasing trend reached a significant level after 2008 (Fig. 6). Moreover, it should be noted that Changchun have an upward trend of reduced fluctuations after 2005.

### Effect of urbanization on the PET in different climate zones

Our results indicated that the most relevant factor to the PET is T-rural (temperature of rural station) across the four cities, which represents background climate conditions of each city (Table 5). In addition to the temperature of rural areas, urbanization also had considerable impacts on PET and the effects of different urbanization variables vary greatly. The GDP is not related to the PET for all four cities, while the secondary industry is positively correlated to the PET in Harbin and Changchun, and the primary industry is negatively correlated with the PET in Changchun. The contribution of these economic factors in Nanjing and Guangzhou is not significant. The effects of population growth on the PET in different cities are also not consistent. The relationship between population and PET is positive in Harbin, while negative in Nanjing. Urban population does not contribute to urban thermal comfort in Changchun and Guangzhou. Overall, our results showed that, among these four typical urbanizing cities, urbanization has a more important role in regulating the urban thermal comfort in cities of humid-continental zone (Dwa) in the northeast China. Urban thermal comfort in cities located in the humid subtropical zone (Cfa) is either irrelevant or negatively affected by urbanization.

**Table 5:** Stepwise regressions of PET and T-rural, population, GDP, primary industry, secondary industry, and tertiary industry of each city.

**Cities**	**Model**	**Unstandardized coefficients**		**Standardized coefficients**	***t***-**Value**	**Sig.**	***R*^2^**
		B	Std. error	Beta			
Harbin	(Constant)	−32.3	6.432		−5.022	0.000	0.905
	T-rural	1.397	0.136	0.729	10.268	0.000	
	Population	2.75E−06	0	0.485	4.708	0.000	
	Secondary Industry	0.001	0	0.255	2.483	0.023	
Changchun	(Constant)	5.769	3.948		1.461	0.170	0.830
	T-rural	0.981	0.163	0.73	6.033	0.000	
	Secondary Industry	0.004	0.001	2.833	3.265	0.007	
	Primary Industry	−0.037	0.014	−2.342	−2.694	0.020	
Nanjing	(Constant)	7.504	6.435		1.166	0.263	0.758
	T-rural	1.375	0.25	0.755	5.508	0.000	
	Population	2.17E-06	0	−0.699	−5.098	0.000	
Guangzhou	(Constant)	3.712	11.264		0.33	0.745	0.233
	T-rural	1.089	0.403	0.483	2.701	0.012	

**Notes.**

The parameter was included in the model when *p* < 0.05, while deleted when *p* > 0.10.

T-rural temperature of rural station

## Discussion

Urban thermal environment has been characterized by higher air temperature, lower relative humidity, and moderation of wind velocity (Cohen, Potchter & Matzarakis, 2012; Mahmoud & Gan, 2018). Previous studies have presented warming trends during rapid urbanization (Ren et al., 2008). Our results also showed increasing of urban temperature during the last 26 years, but the warming trend in our study is not as significant as other studies (Amin et al., 2018; Jacobs et al., 2013). This may because we chose the average value of daily mean temperature in summer days in our study. Our study also showed significant decreasing of wind velocity in Harbin and Changchun, which is similar to many other studies (Yan et al., 2016). However, Guangzhou and Nanjing showed increasing of wind velocity. It could be explained by their local climate conditions that Nanjing and Guangzhou in southeast China are strongly influenced by land-sea interactions for summer (Cao et al., 2018). Urban temperature alone could not adequately represent urban thermal environment (Matzarakis, 2006; Matzarakis & Endler, 2010). The temporal changes of urban temperature and urban thermal comfort are quite different. Our studies showed that PET exhibits more rapid change than urban air temperature during rapid urbanization. In this study, Harbin has a significant upward trend in Ta at the 5% level, but the PET and urban discomfort days in Harbin present significant upward trends at a 1% level. That is because urbanization decrease the relative humidity and wind velocity in addition to warm climate. Therefore, urban thermal comfort has a more significant increasing trend than temperature and can be a better indicator to represent urban thermal environment during rapid urbanization.

Previous studies have observed increasing of heat waves in recent decades and predicted constantly increasing in the future (Fischer & Schär, 2010). Our study also showed an increasing of summer urban discomfort days during the past decades especially in Northeast China. Due to global warming and steady urban expansion, the high PET (PET > 40 °C) would occur more frequently in the future, resulting in a higher probability of the severest overheating problem in China. This increase of thermal heat will potentially cause severe health problems and increased number of illnesses and deaths (Huang et al., 2018).

Climate vulnerability could vary at the regional level, depending on the regional temperature (Zhao, Gerety & Kuminoff, 2018). Studies have shown significant contributions of local background climate to urban heat islands (Cao et al., 2018; Zhao et al., 2014). Our findings are in line with former studies, that local climate conditions have the most substantial contribution to urban thermal comfort. Urban population also affected urban thermal comfort. The growing concentration of urban population is the most basic connotation of urbanization, and it causes increasing artificial heat emissions. The heat generated by the human body and people’s daily life (such as air conditioning and cars) can influence urban thermal environment. The secondary sector of the economy includes industries that consume large quantities of energy and require factories and machinery to convert raw materials into goods and products. They also produce waste heat causing environmental problems. Our results showed that secondary industry could affect urban thermal comfort in Harbin and Changchun. The average summer temperature in Changchun and Harbin was relatively low compared to Nanjing and Guangzhou, which made the influence of waste heat released by the secondary industry on urban thermal comfort more apparent. Therefore, the PET of cities in Northeast China is more susceptible to urbanization. Different with Harbin and Changchun, the secondary industry had no relationship with urban thermal comfort in Guangzhou and Nanjing, which may because of their background climatic conditions. Due to the geographical condition, land-sea interactions in Guangzhou and Nanjing are relatively modest, in terms of the transport of cool and moist air from the nearby sea, near-surface warming, and drying effects (Cao et al., 2018). Besides, China has strengthened environmental governance in recent years, especially in South China, that may alleviate the changes of the urban thermal environment. The regional variation of urbanization influence on urban climate and thermal comfort may be the results of the variation of environmental efficiencies. Nanjing and Guangzhou are located in the two largest river deltas. They have a better regional economic development as well as an ecological environment (Yu & Wen, 2010). However, cities in the Northeast China have relatively weak environmental sustainability (Yu & Wen, 2010). Therefore, Nanjing and Guangzhou have higher environmental efficiencies in contrast to those northeast cities. Changes of urban thermal environment caused by urbanization need to be taken seriously, especially in the cities in the Northeast China. Some regulations for reforming technology or other measures in these cities such as intensively recycling waste and subsidizing sewage disposal plants have played an important role in contributing to emission reduction. This also helps to mitigate the increasing trends of PET in those cities.

This paper explored the relationship between urban thermal comfort and urbanization variables in four Chinese cities. Due to the limitation on collection of long-term urbanization information, this study only selected four typical rapidly urbanizing cities from two different climate zones as research area. Further study should aim to investigate urban thermal environment with more diverse climate conditions.

## Conclusions

Our study explored the changes of urban thermal comfort in four Chinese cities (Changchun, Harbin, Nanjing and Guangzhou) with different climate from 1990 to 2015 and examined the effects of urbanization on urban thermal comfort. Several out comings have been found from our study. Urbanization can lead to increasing of urban temperature, and decreasing of relative humidity and wind velocity. Such effect is inconsistent among cities. The thermal comfort has undermined in Harbin, Changchun, and Guangzhou. Summer urban discomfort days haven lengthened during these 26 years, especially in Harbin. The most important contribution to the changes of urban thermal comfort is the background climate condition, followed by urban population and the secondary industry. Compared with southern cities, the thermal environment in northern cities such as Harbin and Changchun is more sensitive to urbanization. Our studies would provide valuable information on the macro-control urban environment and exert more sustainable urban planning and management strategies.

##  Supplemental Information

10.7717/peerj.8026/supp-1 Supplemental Information 1 Meteorological data from 1990 to 2015

## Additional Information and Declarations

### Data Availability

The following information was supplied regarding data availability: The raw measurements are available in the Supplemental File.
